# Role of IRE1α/XBP-1 in Cystic Fibrosis Airway Inflammation

**DOI:** 10.3390/ijms18010118

**Published:** 2017-01-09

**Authors:** Carla M. P. Ribeiro, Bob A. Lubamba

**Affiliations:** 1 Marsico Lung Institute/Cystic Fibrosis Research Center, The University of North Carolina at Chapel Hill, Chapel Hill, NC 27599, USA; 2 Department of Medicine, The University of North Carolina at Chapel Hill, Chapel Hill, NC 27599, USA; 3 Department of Cell Biology and Physiology, The University of North Carolina at Chapel Hill, Chapel Hill, NC 27599, USA

**Keywords:** cystic fibrosis, airway inflammation, airway epithelia, airway macrophage, innate immunity, UPR, IRE1α, XBP-1, CFTR

## Abstract

Cystic fibrosis (CF) pulmonary disease is characterized by chronic airway infection and inflammation. The infectious and inflamed CF airway environment impacts on the innate defense of airway epithelia and airway macrophages. The CF airway milieu induces an adaptation in these cells characterized by increased basal inflammation and a robust inflammatory response to inflammatory mediators. Recent studies have indicated that these responses depend on activation of the unfolded protein response (UPR). This review discusses the contribution of airway epithelia and airway macrophages to CF airway inflammatory responses and specifically highlights the functional importance of the UPR pathway mediated by IRE1/XBP-1 in these processes. These findings suggest that targeting the IRE1/XBP-1 UPR pathway may be a therapeutic strategy for CF airway disease.

## 1. Introduction

Cystic fibrosis (CF) airway disease is characterized by a chronic and robust inflammatory state often termed “hyper-inflammatory”. In CF airways, the functional absence of the CF transmembrane conductance regulator (CFTR) results in airway surface liquid dehydration, collapse of the cilia, adherence of thickened mucus to airway surfaces, and persistent airway infection leading to chronic inflammation [1,2,3,4]. In addition to decreasing the mucociliary clearance, CFTR mutations affect other innate defensive airway functions. For example, CFTR mutations can impact the internalization of bacteria such as *Staphylococcus aureus*, *Burkholderia cepacia*, *Pseudomonas aeruginosa*, and *Haemophilus influenza*, and alter the release of inflammatory mediators by bronchial epithelial cells [5,6]. The role of the airway macrophage, a key cell involved in innate defense, has largely been overlooked in CF pathophysiology. However, macrophage dysregulation in CF airways could impair resolution of inflammation via an inability to act as a suppressor cell, leading to chronic airway inflammation [7,8].

The endoplasmic reticulum (ER) is a pivotal compartment responsible for many cellular functions. For example, the ER is a site for protein synthesis, folding and post-translational modifications [9]. In addition, the ER is the largest calcium (Ca^2+^) store and ER signaling plays a key role in the synthesis of cholesterol, other lipids, and steroids. Because of its central role in protein synthesis, the luminal ER environment is susceptible to protein misfolding, accumulation, and aggregation [10]. Many physiological conditions and pathological perturbations disrupt protein folding in the ER lumen, causing ER stress, which activates a signaling network known as the unfolded protein response (UPR) [11,12,13,14]. In higher eukaryotes, the UPR is mediated by the activation of three ER stress transducers: IRE1 (inositol-requiring transmembrane kinase/endonuclease-1); PERK (PKR-like ER kinase); and ATF6 (activating transcription factor 6) [12,15,16]. These three arms of the UPR regulate the expression of specific transcription factors, which are responsible for a variety of UPR-mediated responses.

In this review, we will provide a brief overview of UPR signaling and then focus on the contribution of the IRE1α/X-box binding protein-1 (XBP-1) branch of the UPR for the regulation of inflammatory responses of CF airway epithelia and airway macrophages (AMs).

## 2. The Contribution of Airway Epithelia to Cystic Fibrosis (CF) Airway Inflammation

The airway epithelium is a structural barrier that regulates water and ion transport, and contributes to the clearance of inhaled substances through mucociliary clearance. In addition, by producing inflammatory mediators and physically interacting with immune cells, airway epithelia regulate both innate and adaptive immunity. Airway epithelial cells are highly dynamic and display a broad spectrum of activities related to inflammation, immunity, host defense and tissue remodeling [17].

The pathophysiology of CF lung disease is the consequence of a cascade of events resulting from CFTR mutations. Defective CFTR function is coupled with sodium hyperabsorption [18,19], which leads to airway dehydration [20] and impairment of the mucociliary clearance [2,21]. As a consequence, dehydrated mucus accumulates on the airway surface, facilitating persistent bacterial infection [22,23,24,25], which results in chronic inflammation [24,26,27,28,29,30,31]. The CF airway epithelium contributes to the airway inflammatory response, as suggested by studies showing persistent activation of nuclear factor-κB (NF-κB) [32], elevated production of pro-inflammatory cytokines, and decreased secretion of anti-inflammatory mediators [32,33,34,35] in CF epithelia.

Inflammation of airway epithelia triggers expansion of the ER Ca^2+^ stores, which contribute to airway inflammation via Ca^2+^-mediated inflammatory responses [36]. In normal airways, this airway epithelial response may represent a general beneficial adaptation to acute airway infection. However, in obstructed CF airways, the ER Ca^2+^ store expansion-mediated robust inflammation may be inefficient in promoting the eradication of chronic infection in thickened mucus and, consequently, may have adverse effects [11]. These previous studies suggested that the increased CF airway epithelial cytokine production resulting from expansion of ER Ca^2+^ stores are a key contributing factor for the inflammatory status of CF airways.

## 3. The Contribution of Airway Macrophages (AMs) to CF Airway Inflammation

AMs represent a first line of defense against inhaled bacterial pathogens [37]. AMs attack foreign substances and infectious agents, and phagocyte degenerated cells or cellular debris [38,39]. AMs participate in innate immune responses by secreting cytokines, chemokines, and growth factors. In addition to their role in the immune response to infection, AMs can be responsible for efferocytosis, the process of engulfing and eliminating apoptotic cells [40]. Dysregulation of AM function can impair resolution of inflammation via an inability to clear dead cells, leading to chronic inflammation.

Heterogeneity and plasticity are important features of macrophages that play a role in the pathogenesis of certain disorders [41]. Macrophages may respond to various stimuli by polarizing into different phenotypes [42]. Based on their cytokine and chemokine expression and other specific markers, macrophages have been divided into two groups: M1 macrophages (classically activated) and M2 macrophages (alternatively activated) [41]. M1 macrophages are characterized by (a) relatively high secretion of pro-inflammatory cytokines, such as tumor necrosis factor-α (TNF-α), interleukin (IL)-1β, IL-6 and IL-8, and chemokines; (b) high expression of major histocompatibility complex (MHC) class I and II antigens; and (c) secretion of complement factors that mediate phagocytosis [42,43]. In contrast, M2 macrophages express high levels of IL-10 and transforming growth factor β (TGF-β), and play a functional role in immune suppression, tumor growth, and tissue remodeling [44]. Therefore, a balanced and coordinated function of M1 and M2 macrophages is necessary during inflammation and its subsequent resolution.

There has been some attempt to study AM polarization in CF patients. Previous studies have suggested that AMs from CF subjects infected with *P. aeruginosa* are changed toward M2 [45,46]. However, studies in CF mice and human models suggest a contribution of M1 to CF lung disease [47,48,49]. Further studies are needed to evaluate the polarization and phenotype of AMs in CF patients and to understand how they contribute to the pathogenesis of the disease. Nevertheless, initial evidence suggests that AMs are functionally abnormal in CF, and they may play a key role in the robust airway inflammatory response of CF patients [50,51].

It has been hypothesized that AM function is dysregulated in CF because patients are unable to clear chronic infections and have worse outcome in *P. aeruginosa* sepsis compared with patients without CF. Indeed, CF AMs appear to have difficulty to properly eradicate bacteria and may produce greater pro-inflammatory cytokines than non-CF AMs [52,53]. Moreover, even after the occurrence of efficient phagocytosis, CF airways still exhibit increased amounts of bacteria [53], suggesting that CF macrophages also have a defective bactericidal activity. However, these studies did not distinguish whether the macrophages had an intrinsically defective function or the function of additional cells involved in bacterial killing was compromised.

It has been suggested that the defective CFTR alters phagosome acidification, which negatively impacts on the ability of macrophages to kill pathogens [54,55,56]. Contrary to this notion, a separate study provided evidence that the phagosomal acidification in macrophages is independent of CFTR [57]. Hence, it is possible that defective CFTR function might contribute to decreased AM-dependent pathogen lysis by an alternative mechanism. For instance, treatment of wild-type monocytes with CFTR_inh_-172 promotes rises in intracellular Ca^2+^ levels [58]. Because intracellular Ca^2+^ mobilization modulates a variety of cellular responses, including gene transcription, these findings suggest that alterations of intracellular Ca^2+^ levels in CF AMs may affect inflammatory gene expression. Additional studies are necessary to address whether mutant CFTR disrupts intracellular Ca^2+^ homeostasis and, if so, whether this couples to increased inflammatory responses.

Previous studies have indicated that bronchoalveolar lavage fluid from young children with CF contains large concentrations of AMs and the monocyte chemoattractant MCP-1 [59,60]. Moreover, it has been reported that the mitogen-activated protein kinase (MAPK) pathway is hypersensitive to stimulation by lipopolysaccharide (LPS) in CF monocytes [61]. Because the MAPK pathway is involved in critical cell functions (e.g., cell differentiation, cell division, cell migration, apoptosis, and cytokine production), alterations in MAPK signaling can have significant consequences for macrophage immune function. It has also been suggested that LPS hypersensitivity in CFTR-deficient monocytes results from ineffective turnover of toll-like receptor (TLR) 4 [52]. Further support for this notion was given by the finding that monocytes of CF children have increased expression of TLR4, in spite of the absence of infection [62]. Thus, the exaggerated inflammatory response observed in CF monocytes exposed to LPS can be associated with an increase of TLR4 expression. Additional studies are necessary to address whether the increased expression of TLR4 contributes to hypersensitivity reactions, as previously noted [63].

## 4. The Central Role of Unfolded Protein Response (UPR) Activation in Inflammatory Responses

**Figure 1:**
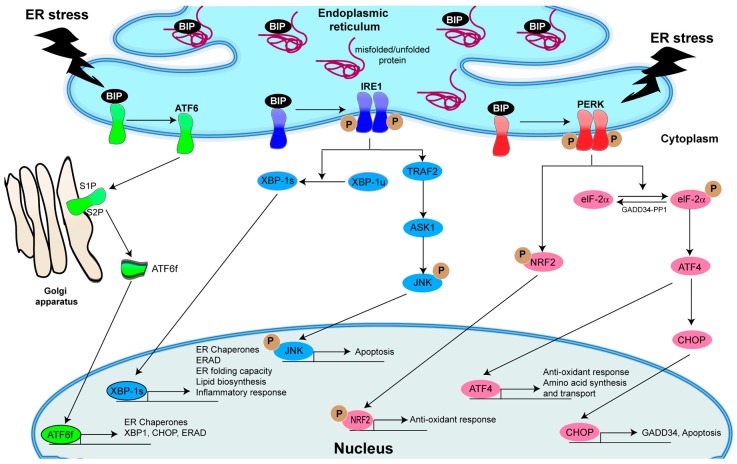
Unfolded protein response (UPR) pathways in mammalian cells. Under non endoplasmic reticulum (ER) stress conditions, BIP (immunoglobulin binding protein) is bound to the ER stress transducers ATF6, IRE1 and PERK, repressing their activation. Following ER stress-induced UPR activation, BIP dissociates from ATF6, IRE1 and PERK, and the following events occur: ATF6 translocates to the Golgi apparatus where it is processed by site 1 protease (S1P) and S2P, resulting in the cleaved active transcription factor ATF6f; in contrast, IRE1 and PERK homodimerize and autophosphorylate, resulting in the generation of the transcription factors XBP-1s and ATF4, respectively. These transcription factors mainly up-regulate the adaptive UPR pathway and normalize ER function via activation of downstream pathways. ATF6f controls the up-regulation of genes encoding ER chaperones, as well as XBP-1, CHOP and ERAD components. XBP-1s is responsible for up-regulating ER chaperones, ERAD components, lipid biosynthesis and inflammatory response genes. ATF4 up-regulates genes involved in anti-oxidant responses and amino acid synthesis and transport. Activation of PERK can also lead to phosphorylation of NRF2, a transcription factor involved in anti-oxidant responses. Under long-term ER stress, the adaptive UPR pathway fails to rescue the cells, and the apoptotic UPR pathways, namely the IRE1–TRAF2–ASK1–JNK or the PERK–eIF-2α–ATF4–CHOP pathways, are induced. Abbreviations: ASK1, apoptosis signal-regulating kinase 1; ATF, activating transcription factor; ATF6f, activating transcription factor 6 fragment; CHOP, C/EBP-homologous protein; eIF-2α, eukaryotic translation initiation factor 2α; ER, endoplasmic reticulum; ERAD, ER-associated degradation; GADD34-PP1, complex of growth arrest and DNA damage-inducible protein 34 and the serine/threonine protein phosphatase 1; IRE1, inositol-requiring transmembrane kinase/endonuclease-1; JNK, c-Jun N-terminal kinase; P, phosphate; PERK, PKR-like ER kinase; NRF2, nuclear factor erythroid 2-related factor 2; TRAF2, tumor necrosis factor receptor-associated factor 2; UPR, unfolded protein response; XBP-1u, unspliced form of X-box binding protein 1; XBP-1s, spliced form of XBP-1.

Numerous environmental conditions may disrupt ER homeostasis, leading to ER stress and UPR activation. The UPR is a sophisticated collection of intracellular signaling pathways that have evolved to respond to protein accumulation and/or misfolding in the ER. In eukaryotic cells, the UPR is activated by the coordinated action of three ER transmembrane stress sensors: (1) IRE1; (2) PERK; and (3) ATF6 (Figure 1). Activation of these sensors results in downstream activation of diverse signaling pathways.

In mammalian cells, activation of IRE1 [64,65], which exists in two isoforms, α (ubiquitously expressed) and β (expressed in gut and airway mucous cells), and ATF6 [66,67,68] increase the ER capacity by transcriptionally up-regulating genes coding for ER chaperone proteins and folding enzymes. Upon UPR activation, IRE1 dimerizes and autophosphorylates, resulting in activation of its endoribonuclease (RNase) activity [67,69]. The IRE1 RNase splices the mRNA of the leucine zipper transcription factor XBP-1 by removing a 26 nucleotide intron, producing a frameshift of the XBP-1 mRNA transcript [70,71]. The resulting spliced XBP-1 (XBP-1s) mRNA is subsequently translated into a potent transcription factor responsible for the up-regulation of genes encoding ER chaperones [70,71,72], protein folding and quality control, ER-associated degradation (ERAD) [73,74], lipid biosynthesis [12,75,76,77], and pro-inflammatory gene production [12,49] (Figure 1). ATF6 is a transmembrane protein that contains a basic leucine zipper (bZIP) transcription factor domain in the cytosolic region [78,79]. Upon activation, ATF6 translocates to the Golgi compartment where it is first processed by site 1 protease (S1P) and then by S2P to produce a cytosolic fragment, which operates as a transcriptional activator of many UPR genes related to protein folding, e.g., ER chaperones, as well as XBP-1 itself [70,80,81]. ATF6 activation is influenced by its oxidation state, glycosylation state, and proteasome-dependent turnover [82,83,84,85,86], and it also plays a role in the ERAD pathway [70,78,87,88]. Thus, some stimuli can favor ATF6 activation more than other pathways. Based on their downstream effects on gene transcription, the activation of the XBP-1 and the ATF6 signaling serve to re-establish the homeostasis of the ER and the secretory pathway.

In addition to its protective function dependent on XBP-1s, activation of IRE1α can trigger apoptosis. For example, under certain conditions of ER stress, activation of IRE1α RNase can also play a role in apoptosis by decaying ER-localized mRNAs, including mRNAs for chaperones [89]. Thus, cellular fate during ER stress depends, in part, on the balance between IRE1α RNase outputs [89]. IRE1α can also activate the apoptotic-signaling kinase-1 (ASK1), which in turn activates the stress kinase Jun-N-terminal kinase (JNK) that induces apoptosis [90] (Figure 1). Bcl-2 and Bim, the apoptosis-inducing substrates of JNK, are inhibited and activated by JNK phosphorylation, respectively [91,92]. Hence, chronic ER stress may change the function of IRE1α from adaptation/protection to producing inflammation and, ultimately, cell death.

Moreover, during prolonged ER stress, the beneficial effects of IRE1α activation may be temporarily and quantitatively delayed, and activation of PERK, the third sensor of ER stress in mammalian cells, can become dominant [67,68,93,94,95] (Figure 1). Stimulation of PERK promotes phosphorylation of the eukaryotic translation initiation factor 2α (eIF-2α), which is responsible for attenuation of general protein synthesis [68]. However, phosphorylation of eIF-2α can also induce selective translation and expression of activating transcription factor 4 (ATF4) [96], a key UPR mediator that induces the transcription of genes involved in amino acid metabolism, oxidative stress, and autophagy [97,98]. Notably, if ER stress is prolonged, ATF4 induces the expression of the transcription factor C/EBP homologous protein (CHOP), also known as growth arrest and DNA damage inducible gene (GADD135) [97,99,100,101], which participates in ER-stress-dependent apoptosis in vitro and in vivo [102,103,104,105]. While CHOP leads to down-regulation of the anti-apoptotic *Bcl-2* gene, it up-regulates pro-apoptotic BH3 domain-only proteins genes such as Bim, and disrupts redox homeostasis, which triggers apoptosis [106]. In cells experiencing chronic ER stress, ATF4 and CHOP proteins function as a heterodimer to promote apoptosis via increased protein synthesis, which enhances protein misfolding, promotes oxidative stress and, ultimately, cell death [107]. Because of the short half-lives of the mRNAs and proteins corresponding to ATF4 and CHOP, only strong and chronic activation of PERK increases the steady-state levels of CHOP to mediate excessive ER stress-promoted terminal UPR [94]. Notably, due to protein phosphatase 2A (PP2A)-mediated serine dephosphorylation of the eIF-2α [108], CHOP is suppressed by TLR signaling during immune responses of macrophages.

Previous studies in mammalian cells revealed that nearly half of the PERK targets are independent of ATF4 [98,109], suggesting the existence of additional PERK-dependent effectors. For example, eIF-2α is also phosphorylated by other kinases that participate in responses to amino acid starvation and accumulation of double-stranded RNA [110]. Thus, cellular responses controlled by eIF-2α are not limited to ER stress. Furthermore, PERK can also phosphorylate the nuclear factor (erythroid-derived 2)-like 2 (NRF2) [111,112], a transcription factor involved in oxidative stress responses [112,113].

Evidence is, therefore, accumulating regarding the role of UPR signaling in inflammation [114,115,116]. Given the central importance of these signaling pathways, it is not difficult to conceive that activation of the UPR plays a central role in the re-establishment of airway epithelial and AM homeostasis during acute inflammation and the long-term outcome of chronic airway inflammatory insults. The sections below focus on the arm of the UPR mediated by IRE1α/XBP-1 because recent findings have revealed crucial functional roles of this pathway in airway inflammatory responses relevant to CF airways disease.

## 5. Role of IRE1α/XBP-1 in Inflammation

**Figure 2:**
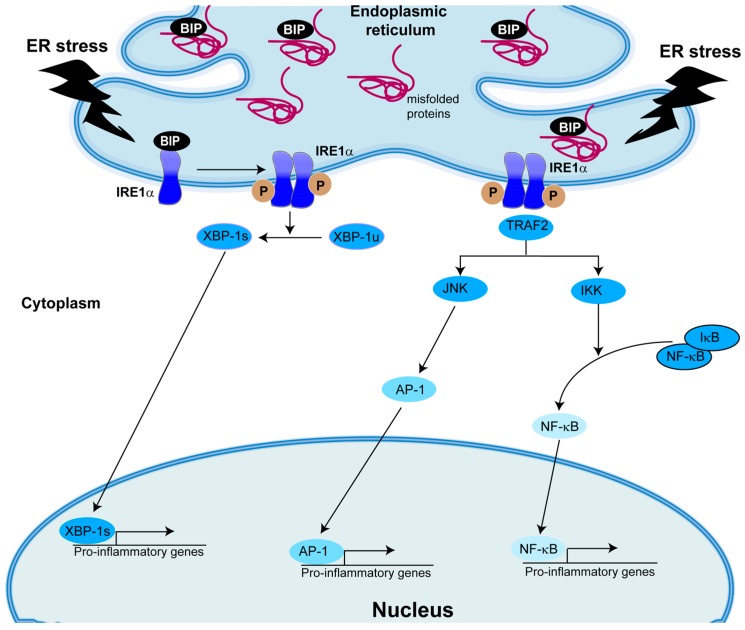
IRE1α-dependent inflammatory signaling pathways. During ER stress, activation of IRE1α can lead to inflammation via the following pathways: (1) Activation of its RNase activity, which processes the mRNA encoding the unspliced X box-binding protein-1 (XBP-1u) to produce an active transcription factor, the spliced XBP-1 (XBP-1s); (2) Formation of an IRE1-TRAF2 complex, leading to activation of JNK and AP1; and (3) Formation of an IRE1-TRAF2 complex, resulting in IκB kinase (IKK) activation, IκB degradation and subsequent activation of NF-κB (nuclear factor-κB). The resulting transcription factors XBP-1s, AP-1 and NF-κB translocate to the nucleus to up-regulate the expression of pro-inflammatory genes.

An overview of the pathways involved in IRE1α-mediated activation of inflammatory gene transcription is provided in Figure 2. Upon activation, IRE1α utilizes its cytoplasmic domain to recruit and activate TNFα receptor-associated factor 2 (TRAF2), which promotes activation of JNK [117,118]. The active JNK subsequently activates the transcription factor activator protein 1 (AP1) [119], which induces the transcription of several inflammatory genes. An additional mechanism responsible for IRE1α-dependent inflammatory responses resulting from its complex with TRAF2 involves the recruitment and activation of IκB kinase (IKK). IKK induces phosphorylation of the NF-κB repressor IκB, resulting in IκB degradation. As a consequence, NF-κB translocates to the nucleus to induce transcriptional regulation of inflammatory genes [120]. Thus, in response to ER stress, the association of IRE1α with TRAF2 can regulate the cellular inflammatory status via activation of distinct transcriptional pathways.

Moreover, in addition to its importance in protein secretion and lipid metabolism, XBP-1s modulates immune responses. For example, ER stress can also be linked to inflammation through activation of TLR [121,122,123,124], since TLR2 and TLR4 ligation has been associated with generation of XBP-1s [123]. In peripheral macrophages, IRE1α activation by TLR engagement is required for optimal and sustained production of pro-inflammatory cytokines (e.g., TNF-α and IL-6) [123]. Notably, these responses appear to be independent from other ER stress markers [123]. These findings are consistent with a previous report showing that prolonged stimulation with LPS inhibits ATF4 activation and CHOP induction [125]. Furthermore, infection of *C. elegans* with pore-forming toxins harboring bacteria leads to the activation of XBP-1 to promote immune defense [126]. In contrast, XBP-1 deficiency markedly increases bacterial burden in mice infected with the TLR2-activating pathogen *Francisella tularensis* [123].

### 5.1. Role of XBP-1s in Human Airway Epithelial Cytokine Secretion Relevant to CF Airways

Previous studies have linked inflammatory responses of human bronchial epithelia (HBE) relevant to CF airways with activation of the IRE1/XBP-1 pathway. For instance, HBE freshly isolated from infected/inflamed CF lungs display increased levels of XBP-1s as compared with non-infected/inflamed normal HBE [11]. Furthermore, native HBE from chronically infected and inflamed CF lungs display up-regulation of calreticulin and protein disulfide isomerase, which are gene targets of XBP-1s [11]. Furthermore, mucosal exposure of primary non-CF HBE cultures to supernatant from mucopurulent material (SMM) from CF airways promotes XBP-1s [11]. These data indicate that the infectious/inflammatory CF airway milieu triggers the UPR mediated by XBP-1s in CF airway epithelia.

Inflamed CF HBE, or non-CF HBE exposed to SMM, exhibit expansion of the ER Ca^2+^ stores, which amplify Ca^2+^-dependent production of interleukin-8 (IL-8) [11,36]. To evaluate the role of XBP-1s in this process, SMM-induced IL-8 secretory responses were assessed in 16HBE14o^−^ cells expressing a control vector or vectors containing XBP-1s or a dominant negative XBP-1 (DN-XBP-1). These studies revealed that cultures expressing XBP-1s exhibited higher basal IL-8 secretory response, as compared with control cultures in the absence of SMM, and potentiation of SMM-induced IL-8 secretion. In contrast, expression of DN-XBP-1 decreased basal IL-8 secretion and blunted SMM-promoted IL-8 secretion [12]. Notably, these studies have also indicated that the increased XBP-1s levels resulting from SMM exposure are responsible for the ER Ca^2+^ store expansion, thereby providing a mechanism for the robust airway epithelial IL-8 secretory phenotype induced by the infectious/inflammatory milieu of CF airways [12]. It remains to be established whether the IL-8 promoter contains a binding site for XBP-1s.

### 5.2. Role of XBP-1s in Human AM Cytokine Secretion Relevant to CF Airways

Pro-inflammatory cytokines such as TNF-α, IL-1β, IL-6 and IL-8 are elevated in the airways of CF patients compared with the airways of healthy controls [127,128,129,130], while the secretion of cytokines involved in resolution of inflammation, such as IL-10, is reduced [128]; importantly, these differences correlate with the number of AMs. Recent studies indicated that primary cultures of human CF AMs exhibit a robust inflammatory phenotype that can contribute to the overall inflammatory status of CF airways [49] based on the following observations: (1) The baseline levels of IL-6 and TNF-α mRNA and their corresponding secreted proteins are higher in primary CF AMs vs. non-CF AMs and (2) the up-regulation of these cytokines after LPS stimulation is greater in CF than non-CF AMs [49].

Importantly, the robust basal and LPS-induced inflammatory responses of primary cultures of human CF AMs correlates with increased levels of XBP-1s [49]. In addition, exposure of primary cultures of human non-CF AMs to SMM from infected/inflamed CF airways, reproduces the robust inflammatory phenotype of CF AMs coupled to larger XBP-1s levels [49]. These findings suggest that the exaggerated inflammation of primary cultures of human CF AMs is mediated, at least in part, by XBP-1s. In support of this notion, the greater inflammatory responses of CF AMs require IRE1α activation-dependent generation of XBP-1s, since treatment with the IRE1α inhibitor 8-formyl-7-hydroxy-4-methylcoumarin (4µ8C) reduces LPS-increased XBP-1s mRNA levels, and TNFα and IL-6 secretion [49]. In addition, overexpression of DN-XBP-1 inhibits LPS-up-regulated XBP-1s and LPS-induced pro-inflammatory cytokine production, whereas overexpression of XBP-1s up-regulates these responses [49].

These studies suggest that activation of IRE1α-dependent XBP-1s is coupled with inflammatory responses in both non-CF and CF AMs, and the higher levels of XBP-1s in CF AMs are proportionate to their robust inflammatory phenotype. The observation that CF AMs exhibit a larger response to LPS-induced inflammation in an XBP-1s-dependent manner supports the notion that XBP-1s is a positive regulator of inflammatory genes resulting from TLR-4 activation in response to acute and chronic bacterial infection in human AMs. These findings offer the proof-of-principle that targeting the IRE1α/XBP-1 pathway may be a therapeutic strategy to decrease the robust inflammatory response of AMs in chronically infected/inflamed CF lungs.

## 6. Cystic Fibrosis Transmembrane Conductance Regulator (CFTR) Mutations Are Not Associated with Activation of XBP-1-Dependent Signaling

### 6.1. Evidence from Airway Epithelia

Previous studies in airway epithelia have suggested that the mutated CFTR controls NF-κB signaling [131,132,133,134,135], although the underlying mechanisms have not been fully established. For instance, the ΔF508-CFTR mutation has been associated with activation of NF-κB in lung epithelial cells [136]. Moreover, it has been suggested that expression of non-mutated CFTR inhibits the airway epithelial production of IL-8 triggered by stimulation of epidermal growth factor receptor (EGFR)-dependent signaling, while loss of CFTR function amplifies EGFR activation-induced IL-8 production [137,138]. These results indicate that the CFTR mutation confers a pro-inflammatory status to airway epithelia. Further support for this notion is given by recent studies showing that knockdown of CFTR increases the expression of NF-κB [139] and cytokines [140].

Notably, while our studies have shown that freshly isolated CF airway epithelia exhibit increased levels of XBP-1s [12,13], earlier work suggested that CFTR mutations are not linked to the higher levels of XBP-1s found in inflamed CF airway epithelia [11]. For instance, CF HBE exhibit increased IL-8 secretion in short-term primary cultures, but this phenotype is lost in long-term cultures [11]. In addition, treatment of long-term CF cultures with SMM mimics the augmented IL-8 secretory response of short-term CF cultures—and this response is linked with induction of XBP-1s by SMM [11]. Subsequent research has suggested that up-regulation of XBP-1s levels are not a consequence of mutant CFTR. For example, dissociation of mutant CFTR from activation of IRE1α/XBP-1s-mediated airway epithelial inflammation has been suggested by a study showing no significant differences in IRE1α activity, intracellular Ca^2+^ mobilization, and IL-8 secretion in CF15 cells overexpressing wild-type or ΔF508 CFTR, evaluated under basal conditions or after *P. aeruginosa* exposure [141]. In contrast, expression of ΔF508 CFTR at high-levels in Calu-3 cells increases XBP-1s [142]. It may be speculated that the differences in activation of IRE1-induced XBP-1s in the latter two studies may be due to differences in specific signaling or differences in the expression levels of ΔF508 CFTR in the cell types investigated. An additional point of consideration is that protein misfolding can lead to ER stress and activation of UPR pathways in airways. It is possible that the high level of expression of misfolded ΔF508 CFTR, which is retained in the ER in airway epithelia, activates the UPR and increases the levels of XBP-1s.

The above studies suggest that ER stress is not triggered in primary CF airway epithelia expressing low levels of ΔF508 CFTR, whereas expression of the mutant protein at high levels in cell lines activates IRE1α and increases XBP-1s. Although the relevance of XBP-1 signaling resulting from exaggerated ER expression of ΔF508 CFTR in cell lines needs to be properly evaluated, the data from primary cultures of inflamed CF airway epithelia expressing endogenous ΔF508 CFTR levels indicate that the CF airway environment, rather than the mutant CFTR, is responsible for promoting ER stress coupled to increased levels of XBP-1s.

### 6.2. Evidence from AMs

Previous studies have suggested the presence of a constitutive/intrinsic mononuclear inflammation in the early stages of CF airway pathogenesis, based on the observation that the number of AMs harvested from young and non-infected CF subjects were elevated when compared with the number of AMs isolated from young control subjects [59]. In addition, the altered CF lung milieu, which is rich in inflammatory mediators [143], polymorphonuclear neutrophil accumulation [144], secreted mucins [143,145] and the associated release of serine proteases [146,147], may persistently activate the TLRs in AMs to optimize pathogen interaction and sensitivity, as well as to stimulate pro-inflammatory pathways. Previous research has indicated that CF AMs exhibit several alterations, including differences in the profile of plasma membrane receptors, impairment in the clearance of particles and dead cells, and larger inflammatory responses and cellular damage [148]. These findings lead to the notion that alterations in the number and function of CF AMs might be caused by the infectious/inflammatory milieu of the CF lung and/or might result from intrinsic, CFTR mutation-related defects [50].

Expression of CFTR has been reported in murine [54,149,150], ferret [151] and human [54,152,153] macrophages, and it has been suggested that CFTR malfunction in macrophages is directly linked with the exaggerated inflammation in CF [52,53,150]. For example, treatment of macrophages with CFTR_inh_-172 promotes increased secretion of pro-inflammatory cytokines [52,54], mimicking the robust inflammatory phenotype of CF AMs. Furthermore, it has been suggested that altered properties of murine CF AMs may contribute to the uncontrolled inflammation in CF lungs [52,150]. For instance, it has been reported that functional CFTR is critical for regulation of phagosomal pH in murine AMs [54], and CFTR-deficient macrophages fail to acidify lysosomes and phago-lysosomal compartments, and display altered bactericidal activity [53,55,154,155]. Furthermore, malfunction of CFTR in human and murine macrophages has been associated with higher secretion of pro-inflammatory cytokines [150,156]. These data suggest that human CF monocytes/macrophages might have an intrinsic defect and abnormal function, which contribute to a pro-inflammatory phenotype.

In contrast, a recent study has shown that the robust inflammatory response of human CF AMs reflects an adaptive response to the chronic luminal infectious/inflammatory milieu of CF airways in vivo and is independent of mutated CFTR, based on the following observations. First, as compared with the levels of CFTR expression in primary HBE cultures, the levels of CFTR expression in primary cultures of human non-CF AMs are close to zero; Second, pretreatment of primary cultures of human non-CF AMs with CFTR_inh_-172 neither increases basal cytokine production nor potentiates LPS-induced cytokine production; Third, exposure of human non-CF AMs to SMM reproduces the robust inflammatory phenotype of human CF AMs coupled to larger XBP-1s levels. Hence, while very low levels of CFTR expression may be important for regulation of other AM functions, as suggested by previous studies [54,55], these findings indicate that the exaggerated inflammation of primary cultures of human CF AMs is not linked to defective CFTR function.

## 7. Conclusions

Evidence indicates that cystic fibrosis (CF) patients have inherited and acquired factors that contribute to abnormal immune regulation, resulting in robust airway inflammation. While previous studies have suggested that cystic fibrosis transmembrane conductance regulator (CFTR) dysfunction confers a pro-inflammatory airway phenotype, other findings, including studies from primary CF airway epithelial cells and macrophages, provide compelling evidence that the exaggerated inflammatory status of CF airways results, instead, from an acquired response to the CF airway milieu. Nevertheless, it is becoming accepted that functional alterations in innate defense of airway epithelia and airway macrophages (AMs) are key contributing factors to CF lung pathology.

There still is a significant knowledge gap regarding the mechanism(s) causing immune dysregulation in CF. However, as discussed in this review, activation of the unfolded protein response (UPR) appears to play a pivotal role in CF airway inflammation. In particular, the UPR pathway mediated by activation of inositol-requiring transmembrane kinase/endonuclease-1 (IRE1α)-dependent generation of XBP-1s has been linked to inflammatory responses of human bronchial epithelia (HBE) and human AMs in translational models relevant to CF airways. Additional studies utilizing in vivo models to investigate the functional role of the UPR in CF airways are necessary. Notably, recent studies with the IRE1β^−/−^ mouse revealed that activation of IRE1β in mucous cells couples to up-regulation of airway epithelial mucin production [15], a key factor in CF airway disease. This function is mediated, at least in part, by X-box binding protein-1s (XBP-1s) [15]. The functional importance of IRE1β signaling for CF airway disease is suggested by its increased mRNA and protein expression in native CF HBE [15]. Although the findings with IRE1β are outside of the scope of this review, they further highlight the importance of IRE1/XBP-1 signaling for the pathophysiology of CF airway disease.

Yet, many questions remain regarding the functional role of IRE1/XBP-1 in CF airway inflammatory responses. For example, it is not known whether IRE1α recognizes different ER luminal substrates as compared with IRE1β, whether they have a similar mode of activation, and whether IRE1β has evolved from IRE1α to exhibit a mucous cell-specific UPR function [15]. In addition, future studies evaluating the role of XBP-1s in specific secretory cell types (e.g., mucin secreting cells) that exhibit a high requirement for increased protein synthesis during inflammation, will be highly useful and may lead to the targeting of this UPR pathway [157] as a novel therapeutic strategy for CF airway inflammatory disease.

## Abbreviations

AMs	Airway macrophages
ATF4	Activating transcription factor 4
ATF6	Activating transcription factor 6
AP1	Activator protein 1
ASK1	Apoptotic-signaling kinase-1
Ca^2+^	Calcium
CF	Cystic fibrosis
CFTR	Cystic fibrosis transmembrane conductance regulator
CHOP	C/EBP homologous protein
DN-XBP-1	Dominant negative XBP-1
EGFR	Epidermal growth factor receptor
eIF-2α	Eukaryotic translation initiation factor 2α
ER	Endoplasmic reticulum
ERAD	ER-associated degradation
GADD135	Growth arrest and DNA damage
IKK	IκB kinase
IL-1β	Interleukin-1β
IL-6	Interleukin-6
IL-8	Interleukin-8
IL-10	Interleukin-10
IRE1	Inositol-requiring transmembrane kinase/endonuclease-1
JNK	Jun-N-terminal kinase
LPS	Lipopolysaccharide
MAPK	Mitogen-activated protein kinase
MCP-1	Monocyte chemotactic protein 1
NF-κB	Nuclear factor κ-light-chain-enhancer of activated B cells
NRF2	Nuclear factor (erythroid-derived 2)-like 2
PERK	PKR-like ER kinase
PP2A	Protein phosphatase 2A
TGF-β	Transforming growth factor β
TLR	Toll-like receptor
TNF-α	Tumor necrosis factor
TRAF2	TNFα receptor-associated factor 2
SMM	Supernatant from mucopurulent material
UPR	Unfolded protein response
XBP-1	X-box binding protein-1
